# Effect of growth temperature on the morphology and phonon properties of InAs nanowires on Si substrates

**DOI:** 10.1186/1556-276X-6-463

**Published:** 2011-07-21

**Authors:** Tianfeng Li, Yonghai Chen, Wen Lei, Xiaolong Zhou, Shuai Luo, Yongzheng Hu, Lijun Wang, Tao Yang, Zhanguo Wang

**Affiliations:** 1 Key Laboratory of Semiconductor Material Science, Institute of Semiconductors, Chinese Academy of Science, Beijing 100083, People's Republic of China; 2 Department of Electronic Materials Engineering, Research School of Physics and Engineering, The Australian National University, Canberra, ACT 0200, Australia; 3 Department of Physics, Tsinghua University, Beijing 100084, People's Republic of China

## Abstract

Catalyst-free, vertical array of InAs nanowires (NWs) are grown on Si (111) substrate using MOCVD technique. The as-grown InAs NWs show a zinc-blende crystal structure along a < 111 > direction. It is found that both the density and length of InAs NWs decrease with increasing growth temperatures, while the diameter increases with increasing growth temperature, suggesting that the catalyst-free growth of InAs NWs is governed by the nucleation kinetics. The longitudinal optical and transverse optical (TO) mode of InAs NWs present a phonon frequency slightly lower than those of InAs bulk materials, which are speculated to be caused by the defects in the NWs. A surface optical mode is also observed for the InAs NWs, which shifts to lower wave-numbers when the diameter of NWs is decreased, in agreement with the theory prediction. The carrier concentration is extracted to be 2.25 × 10^17 ^cm^-3 ^from the Raman line shape analysis. A splitting of TO modes is also observed.

**PACS: **62.23.Hj; 81.07.Gf; 63.22.Gh; 61.46.Km

## Introduction

Semiconductor nanowires (NWs) have been intensively studied in the last decade due to their novel physical properties and potential applications in high-performance devices, such as field-effect transistors, lasers, photodetectors, and photovoltaic devices [1-5]. Such NWs are usually grown through vapor-liquid-solid mode where metal nanoparticles (Au, Ni, or other metals) act as catalysts [6-9]. However, for certain materials the metal catalysts can result in unintentional incorporation into pure crystalline NWs, which causes serious problems for materials doping and limits their device applications. In order to avoid the contamination from Au and other metal atoms, it is highly preferred that NWs can be grown without catalysts.

On the other hand, one of the most attracting features of NWs is that lattice mismatch or strain in NWs can be significantly relaxed due to their high surface/volume ratio and small lateral size. This can be used to realize one of the dreams in semiconductor community--integration of III-V semiconductor on Si platform [10,11], which presents a big challenge due to the significant lattice mismatch and differences in coefficient of thermal expansion between Si and III-V materials. The integration of III-V semiconductor on Si will allow people to take advantage of both the key features of Si like low cost and mature processing technology and those of III-V semiconductor like direct bandgap and high-quality heterostructure growth. Among the III-V semiconductors, InAs NWs possess excellent electron transport properties such as high bulk mobility, small effective mass, and low ohmic contact resistivity, which can be used for preparing high-performance electronic devices such as high mobility transistor [12,13].

Though some work has been done on catalyst-assisted InAs NWs [8,14], little work has been devoted to catalyst-free InAs NWs, especially on Si substrates [5,15,16]. In this paper, we present a study on the catalyst-free synthesis and phonon properties of InAs NWs on Si substrates. By varying the growth temperature, InAs NWs with different diameters were grown on Si substrates. The phonon properties of the InAs NWs are investigated using Raman scattering characterization. The effects of growth temperature on the frequency shift of longitudinal optical (LO), transverse optical (TO), and surface optical (SO) modes are analyzed. Furthermore, a splitting of TO modes also is observed and discussed.

### Experimental details

Vertical InAs NWs arrays were grown on n-type Si (111) substrates in a close-coupled showerhead metal-organic chemical vapor deposition (MOCVD) system (Thomas Swan Scientific Equipment, Ltd., Cambridge, UK) at a pressure of 100 Torr. Trimethylindium (TMIn) and AsH_3 _were used as precursors and ultra-high purity H_2 _as carrier gas. First, Si substrates were cleaned (ultrasonicate in trichloroethylene, acetone, isoproponal, and deionized water sequentially) and etched in buffered oxide etch solution (BOE, six parts 40% NH_4_F and one part 49% HF) for 30 s to remove the native oxide, and then rinsed in deionized water for 15 s and dried with N_2_. Then, the substrates were loaded into the MOCVD chamber for growth. The substrates were heated up to the growth temperature ranging from 530°C to 570°C, and after 5-min stabilization time, the growth was initiated by simultaneous introducing TMIn (2 × 10^-6 ^mole/min) and AsH_3 _(2 × 10^-4 ^mole/min) into the reactor chamber for 7 min. After the growth, InAs NWs were cooled down with the protection of AsH_3 _flow. To obtain more understanding about the controlled growth of catalyst-free InAs NWs on Si, InAs NWs were grown at various temperatures ranging from 530°C to 570°C, *i.e*., 530°C for sample A, 550°C for sample B and 570°C for sample C. The morphology of InAs NWs was characterized by field emission scanning electron microscopy (S-4800, Hitachi, Tokyo, Japan) and high-resolution transmission electron microscopy (HRTEM, Tecnai F20, 200 keV; FEI, Eindhoven, Netherlands). Raman scattering measurements were performed in backscattering geometry at room temperature with a Jobin Yvon HR800 confocal micro-Raman spectrometer (Horiba Ltd., Longjumeau, France). Scattering configuration  () was adopted. The samples were excited by the 514.5 nm line of an Ar-ion laser to a 1 µm spot on the surface with an excitation power of 0.05 mW.

### Results and discussion

**Figure 1:**
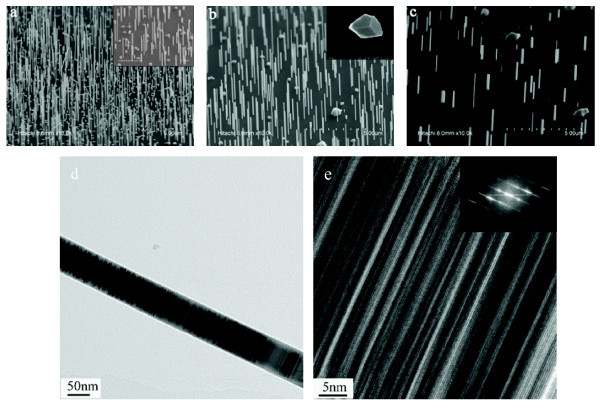
**FE-SEM (45° tilted view) and TEM images of the InAs nanowires grown for 7 min on Si(111) substrates**. Nanowires were (**a**) grown at 530°C (sample A), (**b**) grown at 550**°**C (sample B), (**c**) grown at 570**°**C (sample C); (**d**) low-resolution TEM image of the nanowire. (**e**) High-resolution image of a portion of the nanowires. The inset of (a) shows a higher magnification image of sample A; the inset of (b) is a top view image; the inset of (e) shows the fast Fourier transform of the selected area on (e), which is viewed along the 0 [1-11] direction.

Figure 1 shows the SEM images of samples A, B, and C. It is observed that vertical and uniform InAs NWs with hexagonal cross sections are obtained in all the three samples. With few exceptions, all InAs NWs are grown along the < 111 > direction, which is perpendicular to Si substrate surface. No large base islands are observed at the base area surrounding NWs' root, which is different from the case of catalyst-assisted growth of NWs where large base islands are usually observed [9]. This suggests a different growth mechanism for catalyst-free InAs NWs compared with catalyst-assisted InAs NWs. According to previous work [5], the large lattice mismatch between InAs and Si could be the driving force for such catalyst-free NW growth. InAs clusters/islands first nucleate in Volmer-Weber mode on Si, where uniform film growth is prohibited due to the large interfacial energy. Then, to relax the strain energy in the system, the InAs material is preferred to grow vertically and form NWs. The few large InAs islands and non-vertical InAs NWs observed in sample A, B, and C can be explained by the reoxidation in the system, which provides nucleation sites and reactant sinks and also assist in the growth of larger InAs islands and non-vertical NWs [5,16].

Table 1 summarizes the statistical size information of InAs NWs in the three samples. With increasing growth temperature from 530°C to 570°C, the average density of InAs NWs decreases from 8 to 4 μm^-2^, while the average diameter of InAs NWs increases from 35 to 70 nm. Meanwhile, the average length of InAs NWs also decreased from 2 to 1.2 μm with increasing growth temperature. The change of NW density with increasing growth temperature has also been observed in InAs NWs on InP (111) B substrates [17]. The NWs density is mainly governed by the nucleation kinetics of clusters on the surfaces, and the NW density *ρ *is determined by the materials deposition rate and growth temperature: *ρ *∝ *R/D*(*T*), with *R *being the material deposition rate and *D*(*T*) being the temperature-dependent coefficient of surface diffusion. Therefore, the NW density will decrease with increasing growth temperature. Such change also indicates that InAs clusters or cluster-related nucleation is initiated at the pre-stages of wire growth. At proper temperatures, these clusters grow anisotropically and form one-dimensional NWs. But at lower temperatures, only a part of the clusters follow the anisotropic growth mechanism, others grow by isotropic expansion resulting in larger InAs islands. Indeed, as shown in Figure 1a more InAs large islands are observed in sample A, where InAs NWs are grown at 530 C. Apart from the decreased NW density, the aspect ratio (length/width) of InAs NWs decreases significantly from 57.1 to 17.1 with increasing temperature from 530°C to 570°C. At higher temperatures, the radial growth on the side facets becomes more significant, leading to the formation of NWs with large diameter and small length, and thus small aspect ratio.

**Table 1:** Growth parameters and morphology statistics of InAs NWs grown in sample A, B, and C.

Sample	Temperature	H_2 _flow rate	*D *(nm)	*L *(μm)	*ρ *(μm^-2^)	*L*/*D*
A	530°C	12 L/min	35	2.0	7-8	57.1
B	550°C	12 L/min	42	1.8	5-6	42.9
C	570°C	12 L/min	70	1.2	3-4	17.1

Morphology statistics: average diameter, average length, and average density.

To study the structural properties of InAs NWs, HRTEM measurements were carried out. Figure 1d shows the typical HRTEM image of InAs NWs (sample B). It is observed that the InAs NW is uniform in diameter. It should be noted that alternative dark and bright contrast bands are observed, which can be attributed to the rotation twins and stacking faults. Figure 1e shows the HRTEM image of sample B with its inset showing the fast Fourier transforms (FFTs) image. The HRTEM image combining with FFT image indicates that the InAs NWs has a cubic, zinc blend structure and grows along the < 111 > direction normal to the Si (111) substrate. Such rotation twins and stacking faults are formed by random stacking of the closest-packed planes during crystal growth, which have also been observed in III-V NWs grown along the < 111 > direction [18,19].

**Figure 2:**
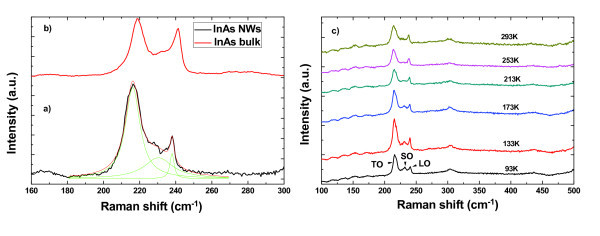
**Raman spectra of InAs nanowires and temperature-dependent Raman shift**. (**a**) Micro-Raman spectra of InAs nanowires with an average diameter of 42 nm. The black line is the recorded data while the lighter colored (green) lines are results from a multiple Lorentzian fit; (**b**) Raman spectrum from bulk (111) InAs; (**c**) Temperature-dependent Raman shift of the TO, SO, and LO phonon mode of InAs NWs.

Figure 2a shows the Raman spectrum of InAs NWs in sample B measured with incident laser beam parallel to the c-axis of NWs. Three main scattering peaks are observed, which are located around 237.9, 230.0, and 216.2 cm^-1^, respectively. To probe the origin of these three Raman peaks, Raman measurements are also performed on bulk InAs (111) substrate for comparison, the spectrum of which is shown in Figure 2b. For bulk InAs materials, two Raman peaks are clearly observed: one is located around 241.0 cm^-1^, the other around 218.7 cm^-1^, which can be attributed to the LO and TO phonon modes of bulk InAs. Therefore, Raman peaks located at 237.9 and 216.2 cm^-1 ^in Figure 2a can be attributed to the LO and TO phonon mode of InAs NWs. Except the downshift of their phonon frequency relative to InAs bulk material, the LO and TO phonon peaks of InAs NWs also show a larger full width at half maximum. To explain such frequency downshift and line-width broadening of LO and TO phonon peaks of InAs NWs, three possible reasons should be taken into account. One is the small lateral size of InAs NWs. According to the "spatial correlation" model proposed by Richter *et al*. [20] and Tiong *et al*. [21], and also generalized by Campbell and Faucher *et al*. [22], the reduction in physical dimension of materials can lead to a downshift of phonon frequency and a broadening of the LO phonon peak due to the strong quantum confinement and the relaxation of *q *= 0 selection rule. However, the diameter of our InAs NWs is very large (> 20 nm) and shows almost no quantum confinement effect, which cannot explain the observed downshift in phonon frequency of LO and TO phonon peaks. Another one is the thermal anharmonicity effect caused by temperature change. Anharmonicity entails the participation of phonons at frequencies multiple of the fundamental in the scattering events [23]. Such anharmonic effects become prominent at higher temperatures due to the larger extent of lattice vibrations, and are irrespective of the longitudinal or transverse character of the phonon modes. Theoretically, an increase in temperature can induce both line-width broadening and frequency downshift of phonon peaks. However, our Raman spectra are measured under a low laser excitation power of 0.05 mW, where the heating effect can be ignored. The last one is the existence of structural defects in NWs. As indicated by the work on GaAs and InAs NWs grown on SiO_2 _and GaAs substrates, defects can cause a frequency downshift and line-width broadening to the phonon peaks [8]. As shown by the HRTEM study, defects like rotation twins and stacking faults exist in the samples, which might relax the *q *= 0 selection rule and lead to the frequency downshift and line-width broadening of the phonon peaks.

As shown in Figure 2, beside LO and TO phonon peaks, there is another phonon peak centered around 230.0 cm^-1^, which can be attributed to the SO phonons. Such SO phonon modes have also been observed in GaP, ZnS, GaAs, and InAs NWs [24-32]. According to the model proposed by Ruppin and Englman [27], the frequency of SO phonon mode in a cylinder NW can be calculated by the expression(1)(2)

Where *ω*_SO _is surface phonon frequency; *ω*_TO _is TO phonon frequency; *ω*_p _is the screened ion plasma frequency, *ε*_0 _and *ε*_∞ _are static and dynamic dielectric constants, respectively; *ε*_m _is dielectric constant of the surrounding medium and *ρ *is expressed as:(3)

where *K*_n_(*x*) and *I*_n_(*x*) (*n *= 0,1) are the modified Bessel functions and *x *= *qr *(*r *being the radius of the NW). For InAs materials, the following parameters are used for the calculation: *ε*_0 _= 13.9, *ε*_∞ _= 11.6, *ε*_m _= 1 (the NWs are immersed in air) [15], *ω*_TO _= 216.7 cm^-1^. The plasmon frequency *ω*_p _can be related to the free carriers concentration (*n*) and effective electron mass of InAs (m* = 0.024*m*_e_) [15],(4)

Vice versa, the free carrier concentration can be calculated if the frequency of SO phonon mode and the size of the NWs are known. Here, the free carrier concentration in sample B is estimated to be 2.25 × 10^17 ^cm^-3^using the measured diameter (42 nm) and SO phonon frequency (230.0 cm^-1^). This result is close to the value obtained through electrical measurements in [5]. This high free carrier concentration in the InAs NWs might be caused by the unintentional doping due to carbon background incorporation [5]. To get more understanding of this SO phonon mode in InAs NWs, temperature-dependent Raman measurements are also performed on the InAs NWs in sample B, the results are shown in Figure 2c. It is observed that the SO phonon peak shifts to lower frequency with increasing the temperature, which is similar to the temperature behavior of the LO and TO mode of InAs NWs, and can be explained by the lattice expansion in NWs. It should be noted that though the SO feature is not apparent at high temperatures (> 173 K) the free carrier concentration should still be around the value (2.25 × 10^17 ^cm^-3^) at low temperatures considering the fact that the free carrier concentration induced by unintentionally doping is much higher than that of intrinsic carrier in InAs materials (~1 × 10^15 ^cm^-3^).

**Figure 3:**
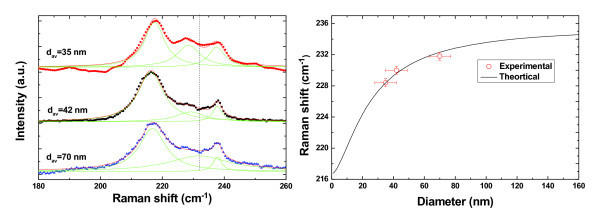
**Raman spectra of InAs NWs measured with different average diameter and theoretical prediction**. (**a**) Raman spectra from InAs NWs with average diameters from 35 nm (red), 42 nm (black), and 70 nm (blue). The lighter colored (green) lines are results from a multiple Lorentzian fit. The vertical line is a guide to the eye. The position of SO phonon down shifts with the decrease in diameter; (**b**) Dependence of the position of the SO phonon from the diameter of the NWs. The points represent experimental data obtained from several measured samples with different average diameters. The line corresponds to the theoretical prediction for cylindrical InAs NWs.

Apart from sample B, Raman experiments are also performed on sample A and C. Figure 3 shows Raman spectra of InAs NWs measured with incident laser beam parallel to the c-axis of NWs at room temperature. As stated above, the phonon peaks on low energy side of LO phonon modes are from SO phonon modes. Obviously, the SO phonon peak shifts toward lower energy side with reducing NWs' diameter. More interestingly, for InAs NWs with smaller diameters (larger surface-to-volume ratio), the SO phonon mode can be more clearly distinguished. These features further indicate that the Raman peak located between TO and LO phonon peaks can, indeed, be attributed to the scattering from surface phonons. According to the model stated above, the phonon frequency of SO mode can be calculated according to the diameter of NWs. Figure 3b shows the calculated phonon frequency of SO mode in InAs NWs with various diameters and the experimentally measured phonon frequency of SO mode of InAs NWs in sample A, B, and C. Obviously, the experimental values agree well with the theoretical values, confirming the SO mode origin of the Raman peak between LO and TO phonon peaks.

**Figure 4:**
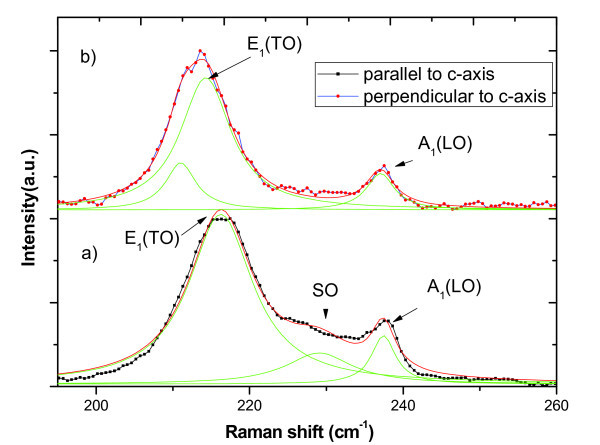
**Raman spectra of InAs NWs recorded parallel and perpendicular to the c-axis**. (**a**) Raman spectra of as-grown vertical aligned InAs NWs (sample B) recorded in backscattering geometry parallel to the c-axis, (**b**) Raman spectra of InAs NWs recorded perpendicular to the c-axis of nanowires. Excitation laser power 0.25 mW, the lighter colored (green) lines are results from a multiple Lorentzian fit.

Figure 4 shows the Raman spectra of InAs NWs with a diameter of 42 nm (sample B) measured with the incident laser beam both parallel  and perpendicular  to the c-axis of NWs. Note that the laser excitation power used for measuring Raman spectra in Figure 4 is 0.25 mW. Compared with the TO peak measured with incident laser beam parallel to c-axis of NWs, the TO peak measured with incident laser beam perpendicular to c-axis of NWs shifts to lower frequency with asymmetric broadening, where a weak shoulder peak appears at the lower energy side of TO mode. This indicates a possible splitting of TO mode, giving rise to A_1 _(TO) mode reported [31]. A more detailed study on the splitting as a function of the NW crystal structure, strain, diameter, and length is currently under way.

## Conclusion

To summarize, the catalyst-free, growth, and phonon properties of InAs NWs on Si (111) substrates are investigated in detail in this paper. Both the density and the length of InAs NWs decrease with increasing growth temperatures, while the diameter of InAs NWs increases with increasing growth temperature, suggesting that the catalyst-free growth of InAs NWs are governed by the nucleation kinetics in the system. The LO and TO mode of InAs NWs both present a phonon frequency smaller lower than those of InAs bulk materials, which is speculated to be mainly caused by the defects in the NWs. Apart from LO and TO phonon modes, a SO mode is also observed for the InAs NWs, the signal feature of which becomes more prominent with reducing the diameter of NWs due to the increased surface/volume ratio. A splitting of transverse optical (TO) modes also is observed.

## Abbreviations

NWs: nanowires; MOCVD: metal-organic chemical vapor deposition; LO: longitudinal optical; TO: transverse optical; SO: surface optical; SEM: scanning electron microscopy; HRTEM: high-resolution transmission electron microscopy.

## Competing interests

The authors declare that they have no competing interests.

## Authors' contributions

TL carried out the experimental analysis and drafted the manuscript. YC carried out the experimental design. WL and XZ participated in the experimental analysis. SL carried out the growth and optimization of InAs NWs. YH participated in the experimental measurement. LW participated in its design and coordination. TY and ZW participated in the experimental design. All authors read and approved the final manuscript.
